# Buccal fat pad excision for cheek refinement: A systematic review

**DOI:** 10.4317/medoral.24335

**Published:** 2021-05-23

**Authors:** Bassel Traboulsi-Garet, Octavi Camps-Font, Marwan Traboulsi-Garet, Cosme Gay-Escoda

**Affiliations:** 1 DDS. Fellow of Master’s degree programme in Oral Surgery and Implantology, Faculty of Medicine and Health Sciences, University of Barcelona, Spain; 2 DDS, MSc. Master’s Degree Programme in Oral Surgery and Implantology. Faculty of Medicine and Health Sciences, University of Barcelona, Spain; 3 Researcher at the IDIBELL Institute.; 4 Associate professor of Oral Surgery and Professor of the Master’s degree programme in Oral Surgery and Implantology. Faculty of Medicine and Health Sciences, University of Barcelona, Spain; 5 DDS. Master’s Degree Program in Orthodontics, Faculty of Medicine and Health Sciences, University of Barcelona, Spain; 6 MD, DDS, MSc, PhD, EBOS, OMFS. Chairman and Professor of Oral and Maxillofacial Surgery, Faculty of Medicine and Health Sciences, University of Barcelona, Spain.; 7 Director of the Master’s Degree Programme in Oral Surgery and Implantology, EFHRE International University/FUCSO, Belize; 8 Coordinator/Researcher of the IDIBELL Institute.; 9 Head of Oral Surgery, Implantology and Maxillofacial Surgery Department of the Teknon Medical Centre, Spain

## Abstract

**Background:**

Buccal Fat Pad (BFP) excision has become an aesthetic surgical procedure. Although this procedure is quite common, it is important to bear in mind that the scientific evidence supporting the efficacy of this treatment is scarce and of low quality. The purpose of this systematic review was to analyse all relevant data to assess the efficacy and safety of BFP excision for improving midface aesthetics.

**Material and Methods:**

A thorough search of MEDLINE (PubMed), Scopus and Cochrane Library databases was conducted. The PICO approach was used where healthy patients seeking cheek slimming and facial silhouette refining undergo BFP excision and were compared before and after surgery in terms of BFP volume reduction, adverse effects and patient satisfaction.

**Results:**

Of the 1,413 references identified, 4 were included in the qualitative synthesis. Only one study reported BFP volume reduction, which was 3.10 mL (95%CI: 2.38 to 3.80; *P* < 0.001), and the mean volume of the excised tissue was 2.74 ± 0.69 mL (range, 1.8-4.9 mL). 84.6% of the patients stated that their facial contour was much better and the remaining 15.4% noticed that the appearance of their cheeks following BFP excision was better. Seven complications were reported in the 134 cheek refinement procedures.

**Conclusions:**

BFP removal has an initially favorable outcome for facial aesthetics and a low postoperative complication rate, however, there are many procedures being performed with poor quality methodology and there is also a lack of published data on its long-term follow-up results.

** Key words:**Buccal fat pad, buccal fat pad excision, surgery, plastic, aesthetics, cheek refinement, facial silhouette refining.

## Introduction

The buccal fat pad (BFP) -also known as Bichat’s ball, Bichat’s fat pad or corpus adiposum buccae- was first anatomically described by Marie-François Xavier Bichat in 1802 as a well-circumscribed mass of adipose tissue located bilaterally in the maxillofacial region (1). Although it was initially considered to be a non-functional structure, studies have shown that the BFP has several significant functions. It plays an important role in masticatory function especially in infants who are suckling. The BPF diminishes in size as the surrounding facial structures develop with the infant’s growth. In adults, the BFP enhances inter-muscular motion and resembles orbital fat in appearance and function (2).

BFP is a very useful structure for reconstructive surgery. The BFP has been widely used as a graft or pedicled flap for the reconstruction of intraoral defects such as oro-antral communication/fistula closure and also for reconstructing maxillary defects (3).

Usually, BPF removal is performed intraorally under local anesthesia. A 2.5-cm incision is made through the mucosa and muscle in the maxillary gingivobuccal sulcus (4-6). While applying external pressure on the skin, the buccal muscle is then dissected and the BFP is exposed. At this point, the protruding portion of the BFP is pulled out, gently teased into the area, clamped at its base, and excised. Finally, an absorbable suture is used to close the wound (2,7). The potential complications may include: hematoma, trismus, infection, facial nerve impairment, parotid duct injury, over-resection, induration, and asymmetry (7).

Although several authors have stated that BFP excision is a simple and safe surgical procedure that is routinely performed, information on its long-term results and complications is scarce. Thus, a systematic review of the existing evidence on this topic may provide new information. Consequently, the aim of the present study is to analyse all relevant data in order to assess the efficacy and safety of BFP excision as an aesthetic procedure for improving midface aesthetics.

## Material and Methods

- Protocol and registration

This paper adheres to the Preferred Reporting Items for Systematic Reviews and Meta-Analyses (PRISMA) declaration (8) and is registered in PROSPERO under number CRD42018101951.

- Eligibility criteria

The predefined study population (P), intervention (I) or exposure (E), comparison (C), outcome parameters (O) and study type (S) (PI(E)COS factors) for eligibility of the studies were:

P: Healthy patients seeking cheek slimming and facial silhouette refinement.

I(E): BFP excision.

C: Preoperative assessment.

O (Primary outcome): BFP volume reduction using an objective method (such as ultrasound imaging) at least 6 months after surgery.

O (Secondary outcome): Adverse events occurring during surgery and/or in the follow-up period.

O (Secondary outcome): Patient satisfaction.

S: Original, randomised and non-randomised clinical trials, prospective or retrospective human case-control or cohort studies.

In order to reliably assess the effect of BFP excision in cases where a combination of procedures was required, the studies had to provide the results of each technique separately. They also had to properly describe the procedures performed.

The review excluded studies that were not published in English and did not include any of the outcomes of interest or with those that had less than 10 patients.

- Search strategy

An electronic search of the MEDLINE (OVID), The Cochrane Library (Wiley), Scopus (Elsevier) and the Web of Science (Thomson Reuters) databases up to May 28, 2019 was conducted in order to identify all relevant human studies without year or language restrictions.

For the PubMed library, the following research terms were applied: (“buccal fat pad”[Title/Abstract] OR “Bichat’s fat pad”[Title/Abstract] OR “bichat’s ball” [Title/Abstract] OR “corpus adiposum buccae”[Title/Abstract]) AND (“excision” [Title/Abstract] OR “bichectomy”[Title/Abstract] OR “removal”[Title/Abstract] OR “cheek refinement”[Title/Abstract] OR “cheek slimming”[Title/Abstract] OR “facial silhouette refining”[Title/Abstract] OR “esthetics”[MeSH Terms] OR “surgery, plastic”[MeSH Terms]). When going through the remaining electronic databases, the key terms used were: (‘buccal fat pad’ OR ‘Bichat’s fat pad’ OR ‘Bichat’s ball’ OR ‘corpus adiposum buccae’) AND (‘excision’ OR ‘bichectomy’ OR ‘removal’ OR ‘cheek refinement’ OR ‘cheek slimming’ OR ‘facial silhouette refining’ OR ‘esthetics’.

Additionally, grey literature was searched on OpenGrey as well as the US National Institutes of Health in order to identify additional potential candidates to be included. The research was completed through a manual screening of the references cited in the selected articles and reviews.

- Selection of studies

Two examiners (B.T.G. and M.T.G.) independently selected the studies in accordance with the inclusion criteria. A third reviewer (O.C.F.) resolved any disagreements.

**Figure 1:**
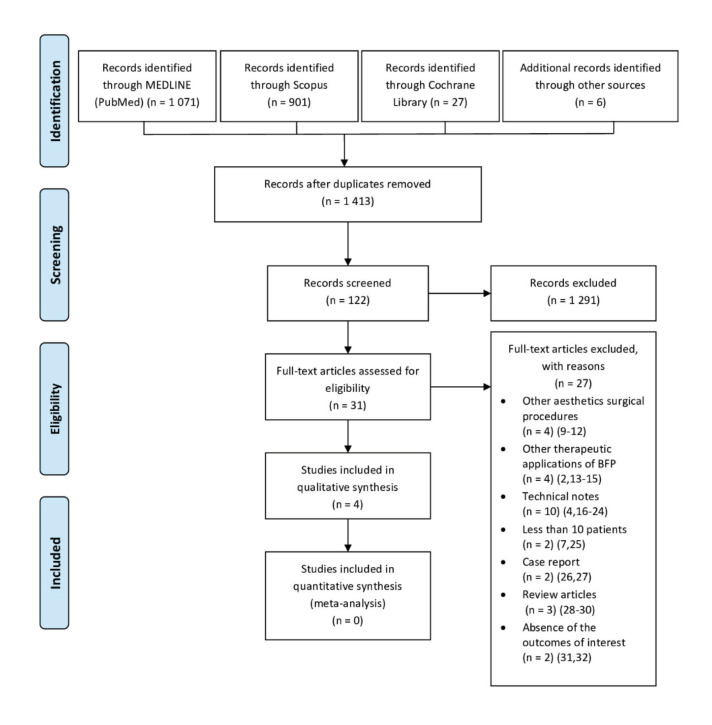
PRISMA flow diagram.

Initially, duplicates or irrelevant publications (based on the title) were excluded, and the abstracts were examined. Finally, the full texts of all the remaining papers were assessed. The studies removed at this stage and the reasons for their exclusion were recorded (Fig. 1).

Authors were contacted for clarification of missing information when it was necessary. When multiple reports on the same patients were identified, only the data with the longest follow-up time was included.

- Data extraction and method of analysis

Two reviewers (B.T.G. and M.T.G.) independently extracted the data using a data-extraction Table. Whenever possible, the following data were retrieved from the selected papers: author(s), year of publication, country of origin, study design and details of the participants, procedure and outcomes.

- Quality and risk of bias assessment

As part of the data extraction process, two reviewers (B.T.G and M.T.G.) independently assessed the risk of bias of the studies included. A modification of the Newcastle-Ottawa Scale (NOS) was used for the assessment of risk of bias in individual observational studies. The following items were evaluated: 1) selection of study groups, 2) comparability of the study groups, and 3) outcome. Each study received a maximum of 9 points.

For RCTs, the Cochrane Collaboration’s tool for assessing risk of bias suggested in the Cochrane Handbook for Systematic Reviews of Interventions (version 5.1.0). The following items were evaluated: 1) random sequence generation, 2) allocation concealment, 3) patient blinding, 4) outcome blinding, 5) incomplete outcome data addressed, and 6) selective reporting. The publications were grouped into the following categories: low risk of bias (possible bias not seriously affecting the results) if all the criteria were met, high risk of bias (possible bias seriously compromising the reliability of the results) and unclear risk if 1 or more criteria were not met and when too little information was available for classification as “high” or “low” risk.

Authors were contacted for clarification of missing or unclear information when necessary.

- Statistical analysis

For dichotomous outcomes, odds ratios (OR) with 95% confidence intervals (95% CI) were used to estimate the effect of exposure. McNemar and exact binomial tests were used to compare the groups. For continuous outcomes, mean differences (MD) and standard deviations (SD) were used to summarise data for each group. The statistical unit was the patient.

The pooled adverse event rate was carried out with Stata14 (StataCorp®, College Station, TX, USA). The 95% confidence intervals (95%CIs) were calculated using an exact binominal approach. A random-effect model analysis was carried out so that a binomial distribution was used to model the within-study variability and the parameters were estimated using a maximum likelihood procedure. A random-effect model effect was chosen because of the statistical heterogeneity between the studies as well as the clinical heterogeneity of the experimental design and sampling.

A pairwise meta-analysis could not be performed because of the heterogeneity of the reviewed studies.

## Results

- Study selection and description

The initial electronic database and gray literature search yielded 1,471 references. After removing duplicate abstracts and assessing both the title and the abstracts, a total of 31 articles were eligible for full-text analysis (Fig. 1). The reviewers’ agreement was 99.73%, with a k index of 0.94 (almost perfect agreement).

After applying the study criteria, 27 publications were excluded because of other associated surgical procedures (9-12), other therapeutic applications of BFP (2,13-15), technical notes (4,16-24), studies with less than 10 patients (7,25), case report (26,27), article reviews (28-30), and absence of the outcomes of interest (31,32), respectively.

Finally, 3 case series studies (5,6,33) and 1 RCT (34) met the inclusion criteria and were selected for qualitative and quantitative synthesis (Fig. 1).

- Risk of bias assessment

All 3 observational studies (5,6,33) were assessed by the adapted NOS. The mean NOS score was 5 (Range: 3 to 8), being the domain “Selection” the highest ranked and the “Comparability” the lowest (Table 1).

The included RCT (34) was considered to have a high risk of bias, mainly owing to selection and detection bias (Table 1).

- Extraction data

The four studies selected included 121 patients, with an age range of 18 to 60 years old and there were 7 times as many females (Table 2) (5,6,33,34). Although all the included reports followed an intraoral approach for removing the BFP, the incision was made in two regions: at bite level (5,33,34) or at maxillary gingivobuccal sulcus (6).

**Table 1:**
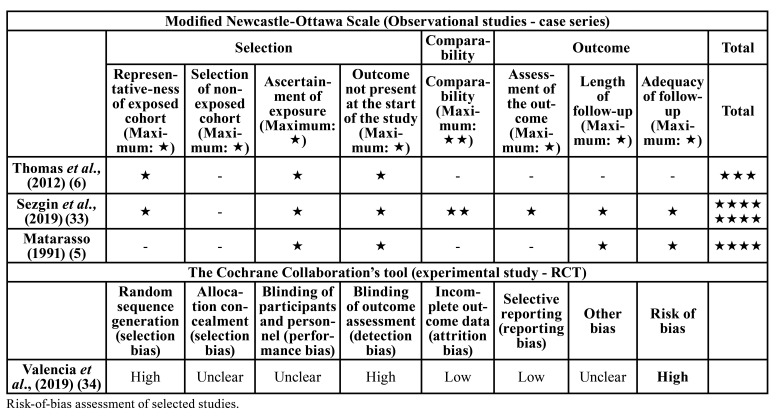
Risk of bias for the included studies.

**Table 2:**
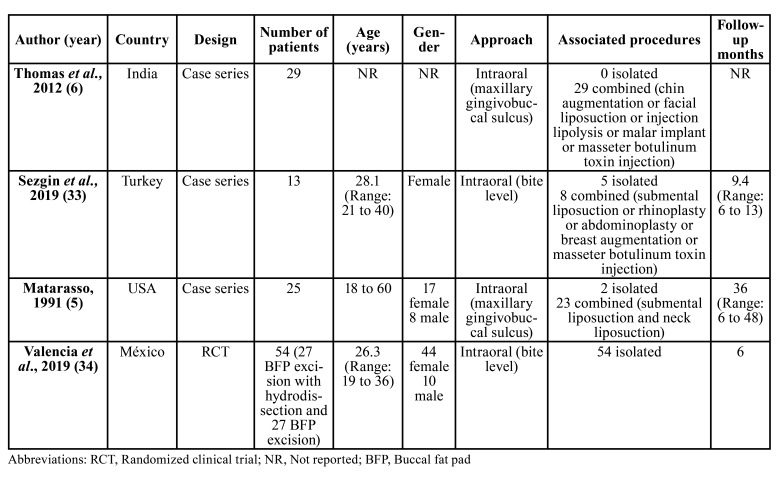
Description of the selected studies.

With regard to anaesthesia regimen, the isolate excision of the BFP was carried out under local anaesthesia or conscious sedation (5,6,33,34). However, general anaesthesia was preferred when extensive associated procedures were performed simultaneously (i.e. submental liposuction or rhinoplasty) (5,33).

Sezgin *et al*. (33) assessed the volumetric changes after the excision of the BFP by means of ultrasound imaging. The authors found a reduction of 3.10 mL (95%CI: 2.38 to 3.80; *P* < 0.001), and the mean volume of the excised tissue was 2.74 ± 0.69 mL (range, 1.8-4.9 mL) 6 months after surgery.

In one study, 84.6% (n=13) of the patients stated that their facial contours had much improved and the remaining 15.4% (n=2) noticed that the appearance of their cheeks following BFP excision had improved (33).

There were seven complications reported in the 134 cheek refinement procedures (weighted mean postoperative complication rate: 3.3%; 95%CI: 0% to 10.5%). The most common complications were trismus (2.24%; 3 cases), transient paralysis of the buccal branch of the facial nerve (1.49%; 2 cases), fever (0.75%; one case) and facial asymmetry (0.75%; one case) and postoperative infections (13.9%). There were no differences were found between the location of the incision (*P* = 0.522) (5,6,33,34).

## Discussion

The present study, which used the recommended methods for systematic reviews and meta-analyses, aimed to analyse all relevant data in order to assess the efficacy and safety of the BFP excision as an aesthetic procedure for improving midface aesthetics, suggesting that the removal of BFP has an initially favorable outcome with regard to facial aesthetics and a low postoperative complication rate.

Nevertheless, the results of the present systematic review should be interpreted with caution since only one RCT comparing two surgical approaches and deemed to have a high risk of bias could be assessed. In this sense, it was somewhat disappointing to find that only a single case series study (33) assessed the volumetric changes after the excision of the BFP. Moreover, it was rather discouraging to observe that we were unable to identify a single observational study with long-term patient follow-up data. Finally, an a priori sample size calculation was lacking in all the selected studies (5,6,33,34), thereby leading to a potentially high type-2 error (failure to reject a false null hypothesis).

BFP excision was firstly described 30 years ago by Epstein (21). The author described a technical note of this procedure indicated for patients with “chubby” cheeks and facial and cervical obesity, which might be poor candidates for rhytidectomy and facelift. These patients might have disappointing results due to persistent fullness of the face despite removal of excess skin. This procedure has recently become quite popular in order to achieve a slimmer lower face silhouette and especially in the medial cheek area (4). However, according to the results of the present systematic review, the scientific evidence supporting the efficacy of this treatment is scarce and of low quality. The procedure, which is performed commonly in the Latin America, lacks scientific evidence on the aesthetic repercussions of this surgical technique. Moreover, in the author’s opinion, the term “bichectomy”, which is commonly used term in the Spanish speaking world, should be considered incorrect. In this sense, BFP excision or removal is the most adequate term since it better represents the nature of the procedure.

Anatomically, the lower face contour is made up of four elements: BFP, the masseter muscle, the mandibular bone, and subcutaneous fat (28). Thus, the BFP has an important role in facial aesthetics and its removal is presented as a technique for improving the appearance of the middle and lower third of the face by highlighting the malar prominence and giving a sculpted facial appearance (4,6,7).

The BFP is described as a round, encapsulated, biconvex structure located in the buccal space which is surrounded by the buccinator muscle medially, the deep cervical fascia and muscles of facial expression anterolaterally, and the parotid gland posteriorly (14,24,34). The body of the BFP is divided into three lobes: anterior, intermediate and posterior; the latter of which has four extensions including the buccal process, pterygopalatine process, pterygoid process, and the temporal process (13). The body and buccal extension, which are the parts that should be removed for midfacial contouring, constitute 55% - 70% of the total volume of the BFP.

Despite the multitude of clinical and aesthetic uses, the proper surgical indications for BFP removal have yet to be fully elucidated (35). Buccal fat pad removal may be considered in for treating buccal lipodystrophy or buccal fat pad pseudoherniation in any age group. It has been stated that the ideal candidate for surgery has strong malar bones which are hidden by prominent cheeks giving the impression of excessive facial roundness and a heavy looking face (5). However, the procedure is contraindicated in patients with hypoplastic malar bones as it can cause unfavourable results secondary to overcorrection (5). Indeed, as BFP involutes with age, its removal may accentuate the appearance of low-lying jowls and expedites facial deformations commonly associated with ageing. Hence, additional procedures such as autologous fat grafts and scaffold injections might be needed in order to rejuvenate the face (30). Due to their limited follow-up, none of the included studies evaluated those features. Because there is limited follow-up information available, none of the studies included evaluated these aspects.

Volumetric evaluations have shown that the BFP begins to grow in childhood and continues to adulthood, increasing from 4000 mm3 to 8000 mm3, and between the age of 20 and 50 drops to 7000 mm3 (35). Surprisingly, in only one of the selected studies was the preoperative BFP volume determined by means of ultrasound imaging (33). In that study, the authors removed a mean volume of 2.74 mL (Range: 1.8 to 4.9) (33). Future research should routinely incorporate preoperative image exams for surgical planning in order to determine the extension and symmetry of BFP and establish a differential diagnosis in cases where there is uncertainty regarding the etiology of cheek fullness (28).

Exposure of the BFP is commonly achieved through an intraoral approach although exposure via a facelift procedure (rhythidectomy) has also been described. When associated with rhythidectomy, impairment of buccal and zygomatic branches of the facial nerve is expected (36). It is therefore our opinion that the intraoral approach for treating buccal fat pad hypertrophy alone is the best option when there are no associated surgical procedures. In these cases, according to Valencia *et al*. (34) BFP excision with hydrodissection should be considered as an effective alternative to the standard procedure because of decreased operative time and surgical manipulation.

BFP removal was performed in isolation in only one of the selected studies (37). In the others, a broad spectrum of associated surgical procedures was required in order to enhance the facial aesthetics of the lower and mid third part of the face, including chin augmentation, facial liposuction, facelift, rhinoplasty, malar implants, masseter detachment and Botulinum toxin injection (5,6,33).

The complication rates of the included studies ranged from 0% to 10.34% of the treated patients. Although most of the reported complications are considered minor, inadvertent surgical manipulation of branches of the facial nerve as well as the parotid duct may also occur. Based on the observations of 19 total hemiface dissections, Hwang *et al*. (37), concluded that because of the presence of anatomical variations, there is a 26.3% chance of injury to the aforementioned structures during the complete removal of the BFP, which may lead to negative consequences ranging from food having a metallic taste, ptosis, and/or tinging or numbness in the face, jaw, or neck (30). Furthermore, it has to be taken into account that since most of the procedures were carried out by experienced clinicians, caution is recommended when extrapolating the results to other clinical scenarios such as general practice. Accordingly, prospective clinical trials should be carried out in order to define the potential pitfalls of the technique. Additionally, it should be taken to account that BFP excision might not be allowed in reconstructive surgery such as oro-antral communication closure in the future (38). For many authors it is an important inconvenience because it becomes an essential resource for many reconstructive surgeries due to its extension, fast epithelization, vascularization, localization and metaplasia process. For all these reasons it becomes a proper flap to cover big defects with a fast healing and low rate of necrosis. Hence, apart from oroantral communications other multiple applications have been widely described in the literature such as: closure of primary cleft palate, coverage of mucosal defects, posttraumatic and congenital facial defects, orthognathic surgery and other oral and maxillofacial surgery procedures (2).

Although BFP removal has an initially favorable outcome in facial aesthetics and a low rate of postoperative complication rate, poor quality methodology and the absence of published data on long-term follow-up results suggests that, BFP resection to aesthetically enhance the mid face should be approached with caution until this data does exist. This only serves to further corroborate the controversy regarding resectioning the buccal fat pad for aesthetically enhancing the mid face.
